# Hyperuricemia Inversely Correlates with Disease Severity in Taiwanese Nonalcoholic Steatohepatitis Patients

**DOI:** 10.1371/journal.pone.0139796

**Published:** 2015-10-06

**Authors:** Jee-Fu Huang, Ming-Lun Yeh, Ming-Lung Yu, Chung-Feng Huang, Chia-Yen Dai, Ming-Yen Hsieh, Meng-Hsuan Hsieh, Ching-I Huang, Zu-Yau Lin, Shinn-Chern Chen, Pi-Jung Hsiao, Shyi-Jang Shin, Wan-Long Chuang

**Affiliations:** 1 Hepatobiliary Division, Department of Internal Medicine, Kaohsiung Medical University Hospital, Kaohsiung, Taiwan; 2 Graduate Institute of Clinical Medicine, Kaohsiung Medical University, Kaohsiung, Taiwan; 3 Faculty of Internal Medicine, College of Medicine, Kaohsiung Medical University, Kaohsiung, Taiwan; 4 Department of Internal Medicine, Kaohsiung Municipal Ta-Tung Hospital, Kaohsiung Medical University, Kaohsiung, Taiwan; 5 Endocrine Division, Department of Internal Medicine, Kaohsiung Medical University Hospital, Kaohsiung, Taiwan; Kaohsiung Chang Gung Memorial Hospital, TAIWAN

## Abstract

**Background & Aims:**

Asians are more susceptible to non-alcoholic steatohepatitis (NASH) as well as metabolic disorder than other ethnicities. We aimed to assess the interaction between metabolic factors and fibrosis in Taiwanese NASH patients.

**Methods:**

A total of 130 biopsy-proven Taiwanese NASH patients (94 males, age = 43.0 ± 13.0 years) were consecutively enrolled. Their demographic, metabolic profiles and histopathological manifestations were analyzed.

**Results:**

Twenty-four (18.5%) NASH patients were non-obese. Thirty-three (25.4%) patients had significant fibrosis (F2) or more: 22 (16.9%) patients were of F2, whilst 11 (8.5%) patients were of advanced fibrosis (F3-4). The prevalence of metabolic syndrome, diabetes and hypertension were 60.8%, 39.4%, and 61.5%, respectively. There was a significant inverse correlation between hyperuricemia and fibrosis stages, ranging from 48.4% of F0-1, 33.3% of F2, and 9.1% of F3-4, respectively (P = 0.01, linear trend). Multivariate logistic regression analysis showed that a decreased serum albumin level (OR = 40.0, 95% CI = 4.5–300, P = 0.001) and normal uric acid level (OR = 5.6, 95% CI = 1.5–21.7, P = 0.01) were the significant factors associated with significant fibrosis.

**Conclusions:**

Hyperuricemia inversely predicts fibrosis stages. Females might carry a more disease severity than males in Taiwanese NASH patients.

## Introduction

In addition to alcoholic induced hepatitis, the importance of non-alcoholic fatty liver disease (NAFLD) has progressively been emphasized in recent decades worldwide. Non-alcoholic steatohepatitis (NASH), as an extreme form of NAFLD, is associated with progressive liver disease and can lead to cirrhosis and ⁄ or hepatocellular carcinoma [1,2]. In the absence of excessive alcohol intake, the close links between NASH and other metabolic disorders, such as obesity, dyslipidemia, hypertension and diabetes mellitus (DM) continue to rise in past decades globally [3]. NASH patients had higher overall mortality compared with controls, and most deaths were due to cardiovascular events [4,5]. In addition, Asian people are more prone to metabolic syndrome (MetS), DM and NAFLD than other races [6]. It is anticipated that NASH will become an increasingly important public health concern in Asia as well as in Westerns.

The risk of fibrosis development in NASH patients should not be overlooked. Around 20% to 30% of patients with NAFLD will develop fibrosing steatohepatitis. Thereafter, 24–30% of patients with fibrosing steatohepatitis may progress to cirrhosis and liver-related deaths, especially in patients with older age, obesity and DM [7–9]. Therefore, early identification of disease progression before the stage of significant fibrosis is critical in the management of NASH. Besides environmental factors, metabolic factors such as obesity or insulin resistance (IR) were involved in the pathogenesis and fibrogenesis of NASH [10]. Most NASH patients have one or more features of the characterized MetS: central obesity, impaired glucose tolerance, hypertension, and dyslipidemia, such as low high-density lipoprotein cholesterol (HDL-C), total cholesterol (TC) and raised triglycerides (TG) [3]. However, the characteristics of Taiwanese NASH patients and the interaction between metabolic factors and disease severity have rarely been investigated.

Consequently, we conducted the current study from a prospective NASH cohort in Taiwan. The aim of the current study was to explore the characteristics of Taiwanese NASH patients. We also aimed to elucidate the extent of the correlation between disease severity and other metabolic abnormalities in NASH.

## Methods

### Study design

This study was an investigator-initiated study of baseline data collected for participants of the clinical trial registered at https://www.clinicaltrials.gov/ct2/show/NCT01068444?term=NCT01068444&rank=1. This prospective study was collaboratively conducted in one medical centre and 2 regional core hospitals in Taiwan from April 2009 to July 2014. The 3 hospitals independently provide the capacity and ability for NAFLD patient care and were referral centres of NAFLD patients from clinics in Southern Taiwan. The ethical committee of the Kaohsiung Medical University Hospital approved the study. Written informed consent for interview, anthropomorphic measurements, blood sampling, and medical record review were obtained from patients prior to enrollment. All subjects underwent a 12-h overnight fast before blood tests, which included fasting plasma glucose (FPG), insulin, TC, HDL-C, low-density lipoprotein cholesterol (LDL-C), TG, uric acid (UA), aspartate aminotransferase (AST) and alanine aminotransferase (ALT) levels. In addition, anthropometric data, which included blood pressure, waist circumference, body weight and height, were measured using standardized techniques. For those without known DM in their past history, they first received a 75-g oral glucose tolerance test (OGTT) and then 2-hour post load plasma glucose level was measured.

### Patient selection

#### Inclusion criteria

Eligible patients were Taiwanese patients aged 18–65 years who satisfied all of the following inclusion criteria: (1) had undergone a liver biopsy within 6 months before entry, the results of which were consistent with NASH, i.e. a combination of steatosis (>5% steatosis), hepatocellular injury and inflammation; (2) displayed an increased serum ALT level, defined as >1.5 times the upper limit of the normal range for at least two measurements within 6 months preceding the study entry; and (3) ethanol consumption of < 20 g/day.

#### Exclusion criteria

Patients were excluded from the study if any of the following criteria existed: (1) laboratory or histologic findings highly suggestive of liver disease of another etiology, such as viral hepatitis, autoimmune hepatitis, primary biliary cirrhosis, biliary obstruction, or genetic liver diseases such as hemochromatosis, alpha-1-antitrypsin deficiency, or Wilson’s disease.; (2) ALT or AST levels greater than 10 times the normal; (3) abnormal total bilirubin or albumin level, prolonged prothrombin time, or platelet count below the lower limit of normal; (4) decompensated cirrhosis (Child–Pugh class B or C) or overt hepatic failure; (5) Treatment with any drugs known to cause hepatic steatosis (i.e., corticosteroids, high-dose estrogens, methotrexate, amiodarone, calcium channel blockers, spironolactone, sulfasalazine, naproxen, or oxacillin) within 6 months prior to the study; (6) psychiatric condition, previous liver transplantation, or evidence of hepatocellular carcinoma.

### Laboratory analyses

FPG, TC, HDL-C, LDL-C, TG, UA, AST and ALT levels were measured on a multichannel autoanalyzer (Hitachi Inc, Tokyo, Japan). Fasting serum insulin levels were measured by radioimmunoassay (Diagnostic Products Co., Los Angeles, CA).

MetS was defined based on the updated National Cholesterol Education Program Adult Treatment Panel III criteria for Asian-Americans, modified by the criteria of obesity proposed for Asians by the Steering Committee of the Regional Office for the Western Pacific Region of WHO as presenting at least three of the following components: 1) waist circumferences >90 cm in men or >80 cm in women; 2) TG >150 mg/dL; 3) HDL-C < 40 mg/dL in men or < 50 mg/dL in women; 4) blood pressure >130/85 mmHg or current use of antihypertensive medications; or 5) FPG>100 mg/dL or on oral anti-diabetic agents or insulin.

The definition of impaired glucose tolerance (IGT) and DM were made according to the American Diabetes Association criteria [11]. For those without known DM in previous medical history, subclinical DM was diagnosed if they met DM criteria (2-h plasma glucose concentration of ≥ 200 mg/dL) with OGTT results.

IR was calculated on the basis of FPG and insulin levels, according to the homeostasis model assessment (HOMA) method [12]. The formula for the HOMA-IR was FPG (mg/dL) × fasting insulin level (*μ*U/mL)/405. IR was considered elevated when it was >2.4 [13].

### Histological analyses

For each patient, a liver biopsy specimen of at least 2 cm in length was taken and fixed in 10% formalin buffer. Biopsy samples were stained with hematoxylin-eosin and the results were then reported by a dedicated liver pathologist blinded to each patient.

#### Steatosis

The extent of hepatic steatosis was graded according to the area occupied by that fatty hepatocytes on light microscopy; none (0–5%), mild (5–33%), moderate (33–66%) and severe (>66%) [14]

#### Histological grading and staging

Histological grading of NASH was made based on histological activity index (HAI) by Knodell et al [15]. It was also assessed on the individual scores for steatosis, inflammation, and ballooning by the NAFLD activity score (NAS) system (0–8) [14]. Fibrosis score for steatohepatitis is determined with the staging from F1 to F4 [16]. Significant fibrosis and advanced fibrosis are defined as F2, and F3-4, respectively.

### Statistical analyses

Frequency was compared between groups using the χ^2^ test, with the Yates correction, or Fisher’s exact test. Results are expressed as mean values ± standard deviation (SD) and were compared between groups using analysis of variance and the Student’s t test, or nonparametric Mann–Whitney U test when appropriate.

The strength of each association is presented as the odds ratio (OR) with 95% confidence interval (CI) and *P* value. All statistical analyses were based on two-sided hypothesis tests with a significance level of p<0.05. Quality control procedures, database processing, and analyses were performed using the SPSS 12.0 statistical package (SPSS Inc., Chicago, IL, USA).

## Results

### Patient characteristics

**Fig 1:**
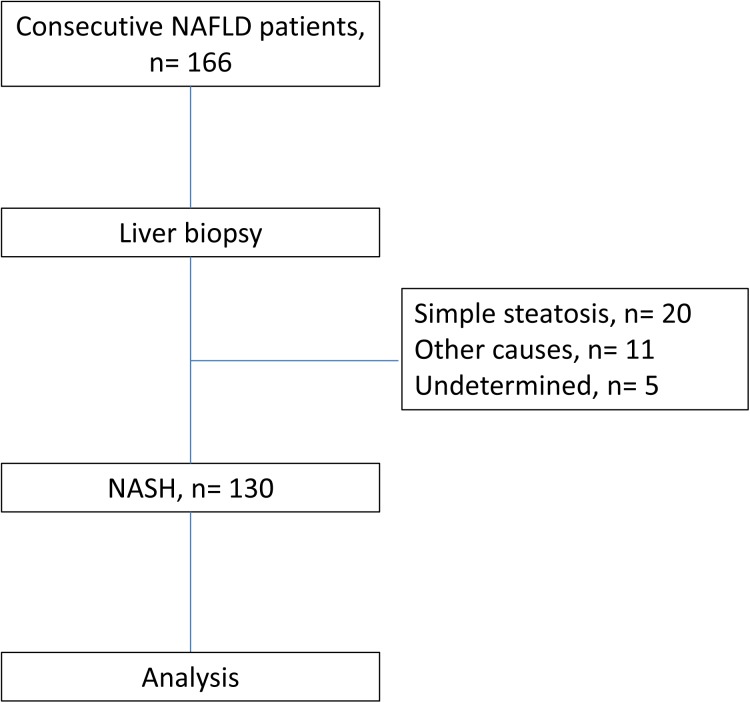
The flowchart of the study.

A total of 166 NAFLD patients were consecutively recruited into histopathological surveillance. Thirty-six patients were excluded, including 20 patients presenting with simple steatosis, 11 patients of other causes of steatosis, and 5 patients of undetermined histopathological manifestations, respectively. A total of 130 NASH patients (94 males, age = 43.0 ± 13.0 years) were enrolled into final analysis (Fig 1). Their demographic characteristics were shown in Table 1.

**Table 1:** Characteristics of the NASH patients according to the presence of obesity. Values expressed as mean ± standard deviation. Parenthesis indicates percentage. NASH, non-alcoholic steatohepatitis; BMI, body mass index; ALT, alanine aminotransferase; AST, aspartate aminotransferase; HOMA-IR, homeostasis model assessment of insulin resistance; HDL-C, high-density lipoprotein cholesterol; TG, triglycerides; γGT, γ-glutamyl transferase; HAI, histological activity index

	Total			P
Characteristic	N = 130	Non-obese N = 24	Obese N = 106	
Age (years)	43.0 ± 13.0	46.7 ± 12.2	42.2 ± 13.1	0.13
Male, n (%)	94 (72.3)	13 (54.2)	81 (76.4)	0.04
BMI (kg/m^2^)	29.0 ± 4.4	23.1 ± 1.9	30.4 ± 3.7	<0.001
ALT (IU/L)	125.4 ± 124.3	129.2 ± 169.1	124.6 ± 112.7	0.87
AST (IU/L)	66.6 ± 55.3	78.3 ± 103.6	63.9 ± 36.5	0.51
A1c (%)	6.4 ± 1.0	6.5 ± 1.2	6.3 ± 1.0	0.38
Fasting glucose level (mg/dL)	107.1 ± 27.0	109.8 ± 37.5	106.5 ± 24.0	0.60
Fasting insulin level (*μ*U/mL)	11.9 ± 8.4	7.9 ± 7.3	12.9 ± 8.4	0.01
HOMA-IR	3.2 ± 2.6	1.9 ± 1.3	3.5 ± 2.7	<0.001
Total Cholesterol (mg/dL)	205.1 ± 36.0	204.6 ± 37.8	205.3 ± 35.8	0.94
HDL-C (mg/dL)	45.1 ± 20.1	44.9 ± 13.2	45.1 ± 21.5	0.15
TG (mg/dL)	164.3 ± 77.7	165.0 ± 85.2	164.1 ± 76.3	0.96
Diabetes, n (%)	31 (23.8)	6 (25)	25 (23.6)	1.0
Hypertension, n (%)	58 (44.6)	8 (33.3)	50 (47.2)	0.26
Uric acid (mg/dL)	6.6 ± 1.6	5.7 ± 1.5	6.9 ± 1.5	0.001
Albumin (g/dL)	4.4 ± 0.3	4.4 ± 0.3	4.4 ± 0.3	0.87
Platelet count (mm^3^)	229.0 ± 58.9	226.7 ± 43.4	229.4 ± 62	0.84
rGT (U/L)	66.2 ± 56.1	75.2 ± 85.2	64.0 ± 46.8	0.38
Ferritin (ng/mL)	314.4 ± 229.7	281.3 ± 196.1	330.9 ± 244.2	0.25
HAI	6.4 ± 2.3	6.0 ± 1.8	6.5 ± 2.5	0.31
Fibrosis, *n* (%)				0.79
F0-1	97 (74.6)	19 (79.1)	78 (73.6)	
F2	22 (16.9)	2 (8.3)	20 (18.9)	
F3–4	11 (8.5)	3 (12.5)	8 (7.5)	

There were 24 (18.5%) non-obese patients with their BMI less than 25 kg/m^2^. Among 106 obese patients, 81 (76.4%) were males, which was significantly greater than their non-obese counterparts (54.2%) (P = 0.04).The 106 obese patients had a higher BMI, a higher fasting insulin level and HOMA-IR, a higher UA level, and a higher NAS compared with their non-obese counterparts.

### Metabolic manifestations

The prevalence of DM and hypertension were 23.8%, and 44.6%, respectively. Among 120 patients who had complete surveillance of each component of metabolic syndrome (MetS), 73 (60.8%) patients met the current criteria of MetS. A high waist circumference (86.3%) was the most frequent component of MetS in the 73 MetS patients, followed by hypertension (80.8%), DM (71.2%), a low HDL-C level (71.2%), and a high TG level (64.4%).

**Fig 2:**
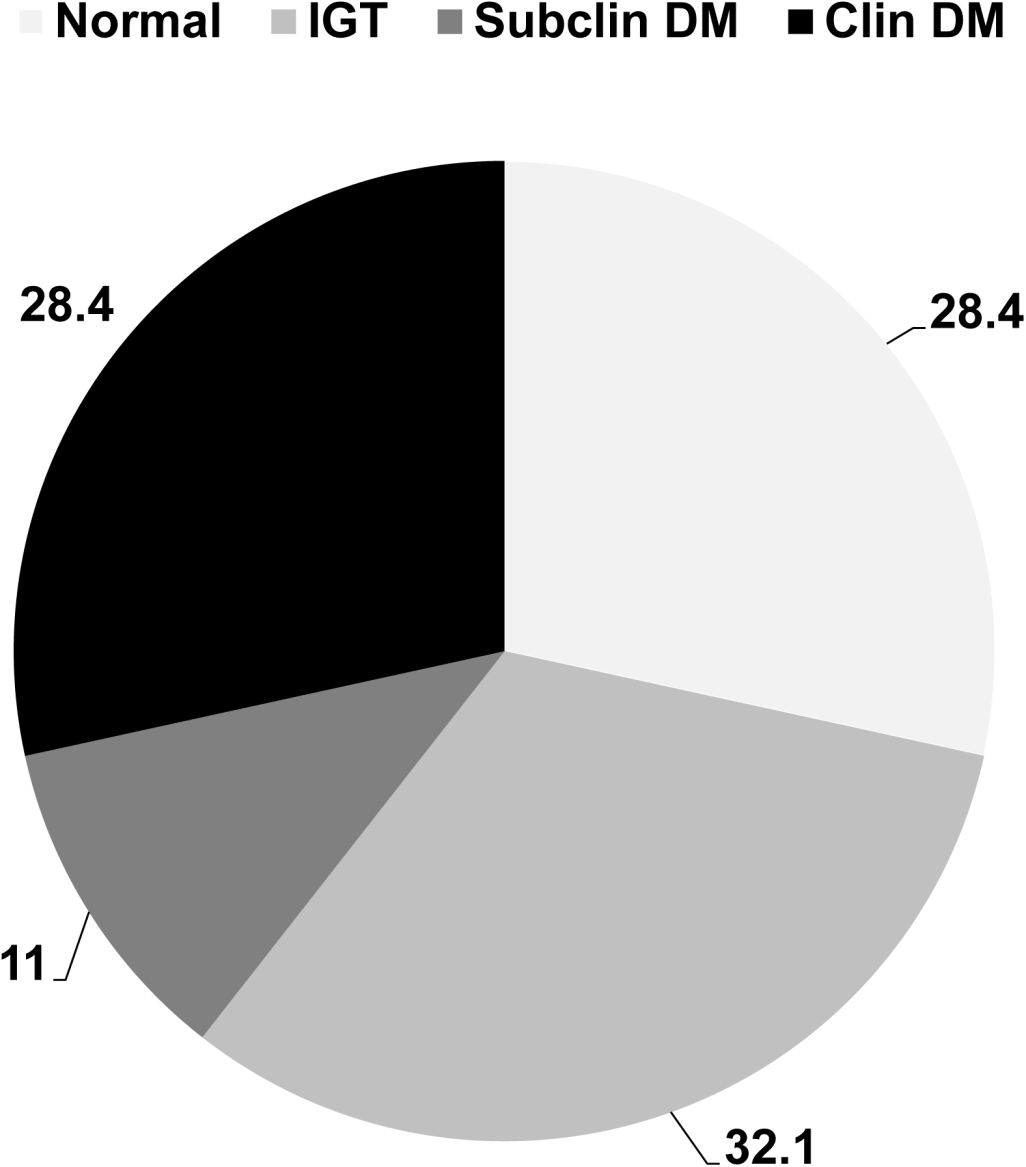
Distribution of glucose abnormalities among NASH patients after oral glucose tolerance test. IGT, impired glucose tolerance; subclin DM, subclinical diabetes mellitus; Clin DM, clinical diabetes mellitus.

There were 31 patients whose DM has been diagnosed before the recruitment. Excluding 21 patients who refused OGTT, a total of 78 patients received OGTT for further validation of glucose abnormalities. There were 12 (11%) patients of subclinical DM, 35 (32.1%) patients of IGT, and 31 (28.4%) patients of normoglycemia, respectively. Overall, 39.4% of patients had DM after OGTT validation (Fig 2).

With current standard measurement procedures, 22 (16.9%) patients who did not have medical history of hypertension were diagnosed to be hypertension. The overall prevalence of hypertension among 130 NASH patients was 61.5%.

### Histopathological manifestations

The HAI and the NAS among the patients were 6.4 ± 2.3, and 5.5 ± 1.8, respectively. Eighty-seven (70.0%) patients had their NAS of 5 or more.

Twenty-two (16.9%) patients were of fibrosis stage 2, whilst 11 (8.5%) patients were of fibrosis stage 3 or 4. There was no significant difference of F2 or F3-4 between obese and non-obese patients. The 33 patients having significant fibrosis and more (≥ F2) carried a higher age, a higher ferritin level, a lower platelet count, and a lower albumin level than their 97 counterparts (F0-1).

**Fig 3:**
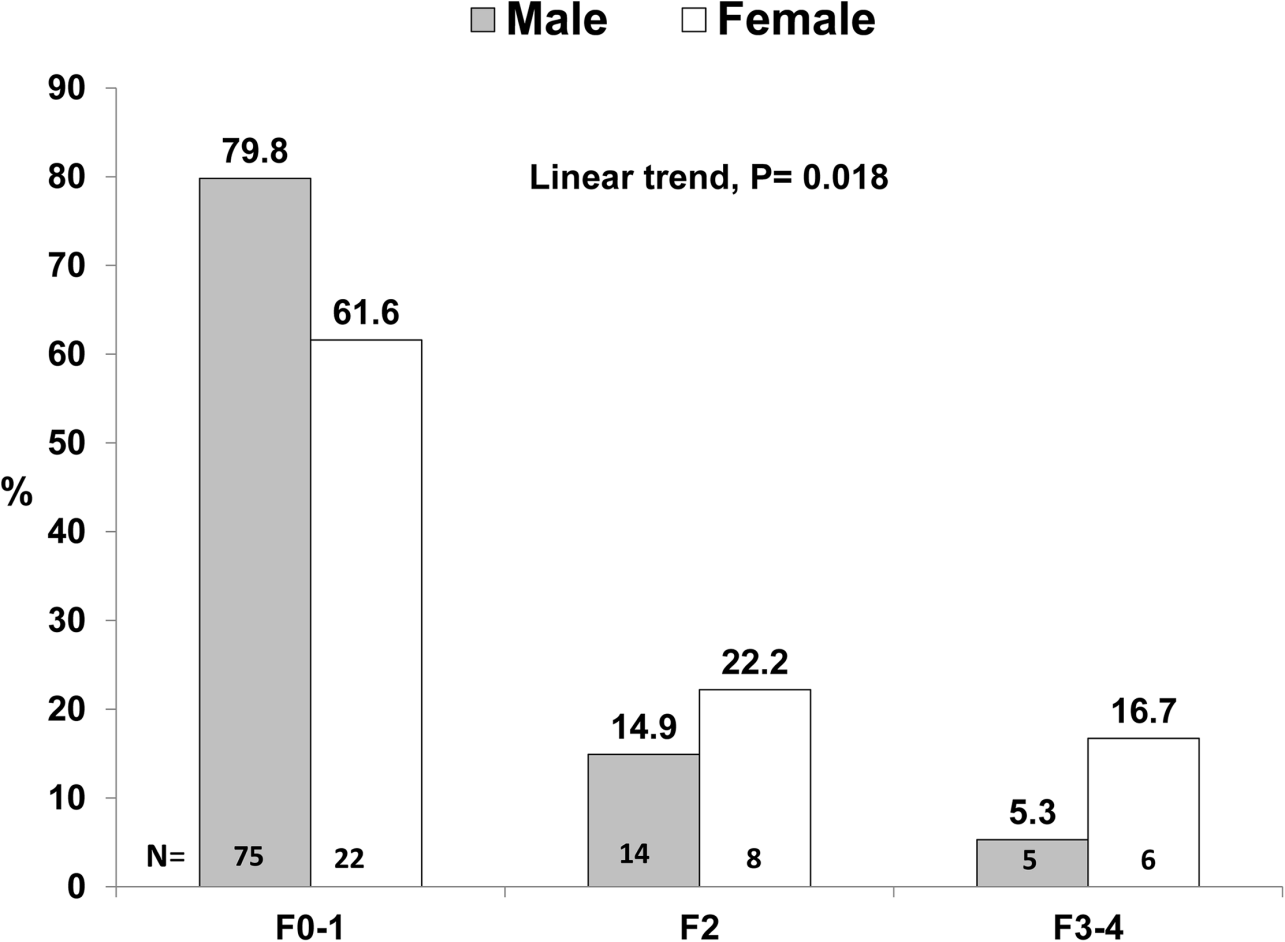
The gender disparity of significant fibrosis (F2) and advanced fibrosis (F3-4) among NASH patients.

The mean age of females was 48.7 ± 11.3 years, which was significantly higher than that (40.8 ± 13.0 years) of males (P = 0.002). There was no significant gender difference of significant fibrosis or more (F≥ 2) among 45 patients aged 50 years or more (40% of males vs 50% of females, P = 0.56). Fourteen (38.9%) female patients had significant fibrosis and more (F≥ 2), and which was significantly higher than that of their 94 male counterparts (19/94, 20.2%). The gender disparity was also observed in patients with advanced fibrosis (F3-4) (16.7% of females vs 5.3% of males) (P = 0.018, linear trend) (Fig 3).

### Correlation between metabolic factors and fibrosis stage

Twenty-one (28.8%) of the 73 patients who met the criteria of MetS had significant fibrosis (F2) or more, which was significantly higher than the 30 patients with 2 components of MetS (23.3%), and the 17 patients without MetS (5.9%) (P for trend = 0.049).

**Fig 4:**
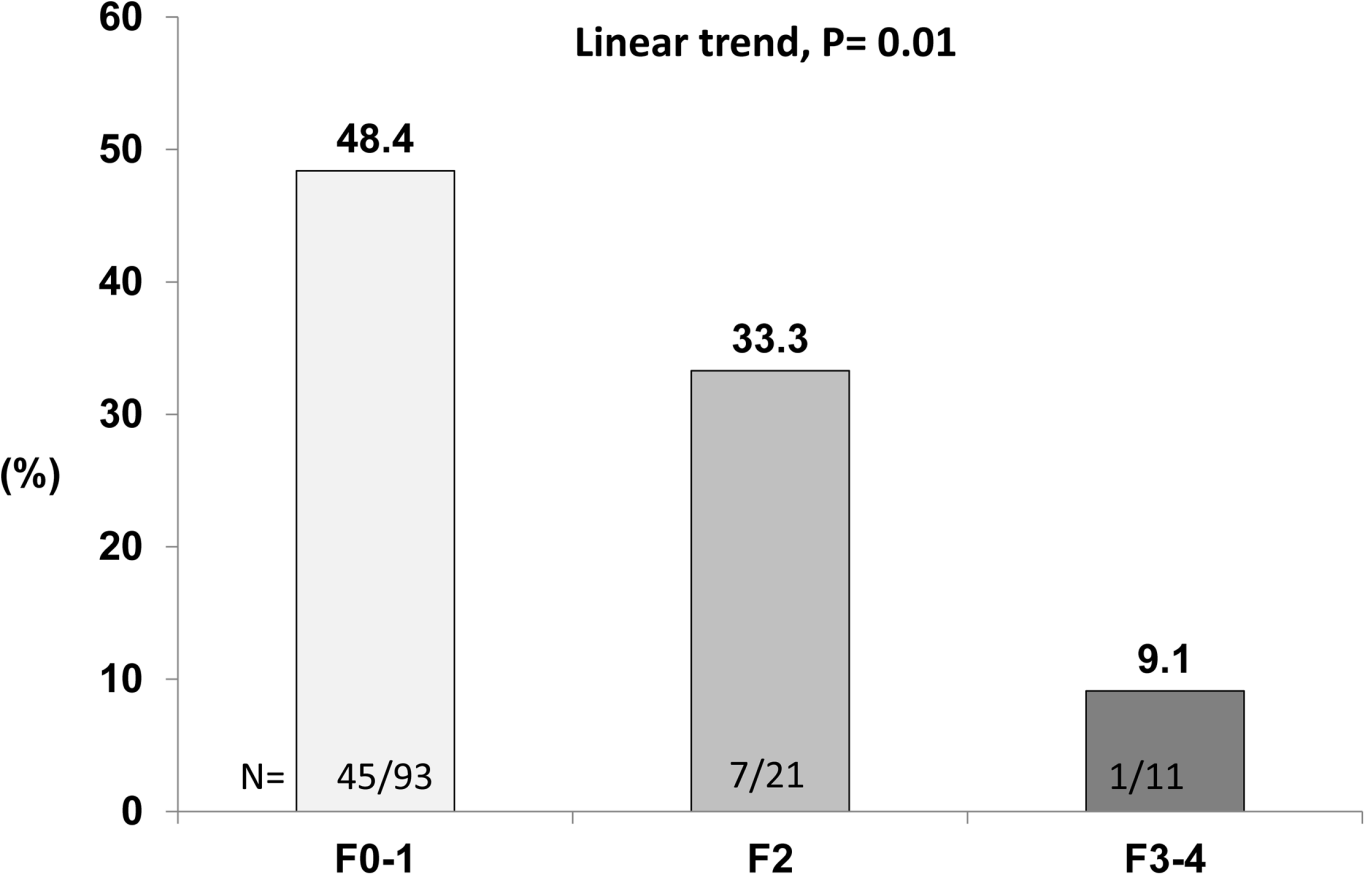
The presence of hyperuricemia according to fibrosis stages among NASH patients.

Among those 125 patients with available UA examination, 47 (52.8%) out of the 89 males carried a hyperuricemia state than the females (6/36, 16.7%, P< 0.001). There was a significant inverse correlation between uric acid level and fibrosis stages, ranging from 7.2 ± 1.3 mg/dL of F0, 6.5 ± 1.7 mg/dL of F1, 6.3 ± 1.6 mg/dL of F2, and 6.0 ± 0.8 mg/dL of F3-4, respectively (P = 0.04) (S1 Fig). The proportions of hyperuricemia among those patients with F0-1, F2, F3-4 were 48.4%, 33.3%, and 9.1%, respectively (P = 0.01 for linear trend) (Fig 4).

Multivariate logistic regression analysis was conducted to assess the factors predicting significant fibrosis. The recruited factors included age, sex, BMI, MetS components, albumin level, rGT level, ALT level, HOMA-IR, and the presence of hyperuricemia (defined as ≥ 7 mg/dL for men and ≥ 6.0 mg/dL for women). A decreased serum albumin level (<3.5 g/dL) was the most significant factor associated with significant fibrosis (OR = 40.0, 95% CI = 4.5–300, P = 0.001), followed by the absence of hyperuricemia (OR = 5.6, 95% CI = 1.5–21.7, P = 0.01).

## Discussion

NASH is a growing liver disease worldwide with the parallel tide of obesity, DM, dyslipidemia, and other associated metabolic disorders. It is plausible that it will become a major health issue in the future with the persistent control of viral hepatitis infections. With the introduction of westernized lifestyle and the increasing frequency of obesity in the Asia-Pacific region, the prevalence of NAFLD/NASH has rapidly increased over the past decades [17]. Our study aimed to elucidate the metabolic characteristics and the interaction with fibrosis in a prospective biopsy-proven NASH cohort in Taiwan. We demonstrated that 18.5% of our NASH patients were non-obese. One-quarter of our NASH patients carried significant fibrosis or more, and the figure was more than 20% even in non-obese patients. A high prevalence of MetS was observed in our cohort and MetS was associated with disease progression in NASH patients. In addition, a lower albumin level and a lower UA level were the factors significantly predicting significant fibrosis in NASH patients. Our study thus provides a comprehensive elucidation of NASH in terms of metabolic and histopathological manifestations and their interaction in Asians.

Several studies among both western and eastern cohorts demonstrate that Asians consistently have a much lower BMI compared to other ethnic groups [18,19]. The relatively lower BMI is not protective in Asians. The rates of hypertension and DM, while somewhat lower, still continue to demonstrate rising trends among Asians [6]. In addition, despite having significantly lower BMI than other ethnic groups, Asians have a surprisingly high prevalence of NAFLD [20]. Previous community-based Asian studies indicated that the proportion of non-obesity in NAFLD patients ranged from 17% to 75% from Asia [17]. Our study from a biopsy-proven NASH cohort further stringently extended the observation that 18.5% of NASH patients were non-obese. Lifestyle changes, environmental and ethnic factors may contribute to the development of NASH in non-obese patients. It may imply that NASH is a disease of underestimation. Of note is that the gender disparity became more indistinct in non-obese patients. Study exploring the genetic and/or epigenetic difference between genders will be needed to further elucidate the novel observation.

Liver fibrosis is the final result of a wide variety of types of liver injury. Despite the absence of established treatment regimens in NASH, the presence of significant fibrosis is an important and early check point, which highly implies that the disease may progress and may result in cirrhosis[21]. The fibrosis staging of NASH in Asians has been reported to be relatively milder than that of Westerns [9,22,23]. The reason for the lower prevalence of advanced disease in Asian NAFLD patients is not fully clear. We observed that 25.4% of NASH patients had significant fibrosis or more, and it reached 20.8% in non-obese patients. Of note is that the presence of significant fibrosis was not significantly different between obese and non-obese patients. Adipose tissue distribution and genetic predisposition may largely contribute into the phenomenon in NASH Asians. It might also imply that to make a stringent surveillance regarding disease progression should be more emphasized in non-obese NASH patients.

IR is the major concealed player of the scenario in NASH. The subsequent and/or concomitant metabolic disorders such as DM and hypertension need to be paid more attention. With current stringent measurement of OGTT, an additional 11% of DM patients and 32.1% of IGT patients were explored. Our results echo previous Asian studies showing that 60% NAFLD patients had been diagnosed to have previously undiagnosed glucose abnormalities by OGTT [24,25]. Our study also observed that an additional 16.9% of NASH patients have been reported to have previously undiagnosed hypertension with standard techniques of measurement. The high prevalence of MetS in the current study was quite consistent with previous Taiwanese study [26]. Taken together, our data indicated that IR-associated metabolic disorders such as DM and hypertension may be underestimated in a certain part among NASH patients. Previous study has demonstrated that postprandial hyperinsulinemia was associated with advanced fibrosis in NAFLD [25]. Therefore, OGTT and a standard approach of blood pressure should be considered in NAFLD patients without prior known DM in order to facilitate early therapeutic intervention [27].

NASH was initially regarded as a disease of middle-aged to older women, and the gender-specific incidence increased with age [28,29]. Recent studies have indicated that the prevalence of NAFLD is higher in men and postmenopausal women. Previous study from the well characterized NASH Clinical Research Network demonstrated that patients with biopsy proven NASH were more likely to be female than male in a roughly 2:1 ratio. It possibly reflected a higher disease burden in women or, alternatively, sex differences among those pursuing and receiving healthcare [30]. However, the gender difference of disease severity has rarely been investigated before. Intriguingly we observed that females had a higher disease severity, i.e., both significant fibrosis and advanced fibrosis. Moreover, the gender difference of significant fibrosis became indistinct among those aged 50 years and more. Apart from different aging process, the gender differences in NASH may be probably explained by gender disparities in body fat distribution, lifestyle, and sex hormone metabolism. Estradiol and estrogen receptors in the liver protect hepatocytes from oxidative stress, inflammatory injury, and cell death, which all contribute to fibrosis. However, this rate of disease progression changes over time in women [31]. Our results may imply that women might have a more disease progression rate than men upon the establishment of NASH from simple steatosis. Further longitudinal study will be needed to elucidate the issue.

UA is the end product of purine metabolism in humans and is a prerequisite for gout development. UA is associated with metabolic abnormalities, increased C-reactive protein concentration and endothelial dysfunction, or even, to risk factors for cardiovascular diseases [32]. By contrast, UA carries an antioxidant protective response against oxidative stress and it UA contributes to > 50% of the antioxidant capacity of the blood [33]. The role of UA in the disease course of NASH remains to be elucidated [34,35]. It is formed by the liver and mainly excreted by the kidneys and intestines. Therefore, the severity of liver damage should be taken into consideration before further clarifying the role of UA in the pathophysiological mechanisms of NASH. Our study demonstrated that there was a significant inverse correlation between UA level and fibrosis stages and the absence of hyperuricemia was predictive of significant fibrosis. The results echoed recent nationwide NAFLD study from Japan showing that UA levels decrease with fibrosis progression in 1,365 biopsy-proven NAFLD patients [36]. However, discordant results also demonstrated that hyperuricemia was associated with disease severity [37]. Difference of hyperuricemia prevalence and disease severity across different ethnicities may largely contribute to the discordant results between Asians and Westerns. The exact role of UA in the disease progression of NASH needs to be determined across different ethnicities, nutritional status and associated metabolic disorders in the future collaborative study. In addition, whether the medicine for DM and/or hyperlipidemia had impact on the results remained to be elucidated.

In conclusion, the current study, on a biopsy-proven cohort, provided the comprehensive features of Asian NASH patients. It showed both the metabolic and histological manifestations as well as their mutual links. Near one-fifth of the NASH patients were of non-obese. The gender disparity became indistinct in those non-obese patients. Females with NASH may have a more disease severity. The exact role of uric acid in the pathophysiological mechanisms of NASH remains to be explored. The results need a further long-term study to validate the manifestations and their interaction in the region.

## Supporting Information

S1 Fig The correlation between serum uric acid level and fibrosis stages among NASH patients. (TIF)
