# Supplementary figures and images for: Hyperuricemia Inversely Correlates with Disease Severity in Taiwanese Nonalcoholic Steatohepatitis Patients
Source: PLoS One. 2015 Oct 6;10(10):e0139796. doi: 10.1371/journal.pone.0139796 (PMC4595446; doi:10.1371/journal.pone.0139796)

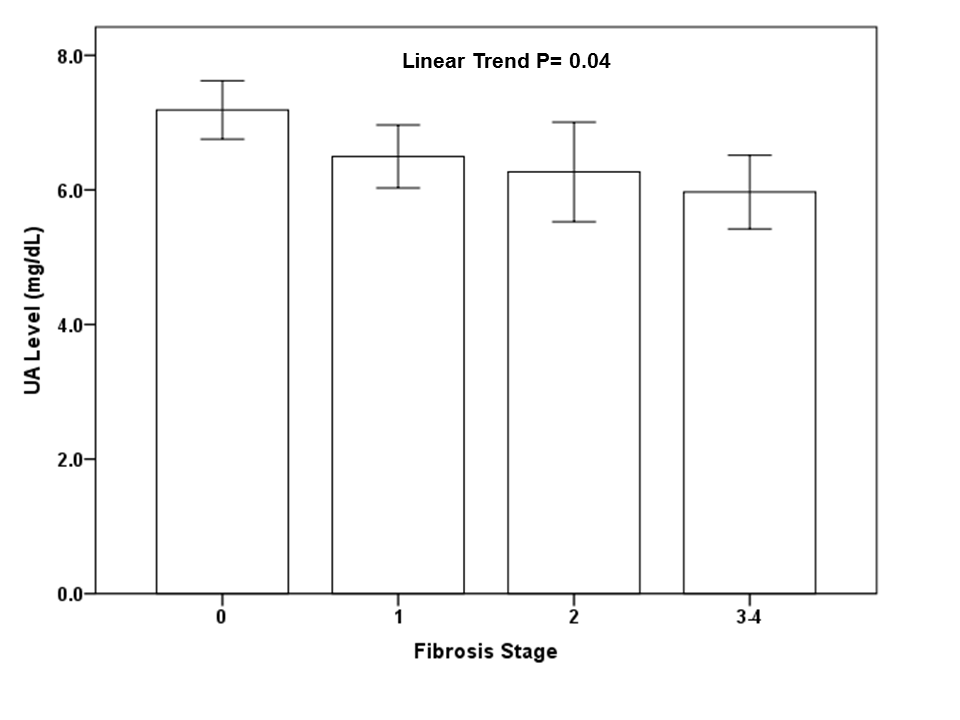

Supplement: S1 Fig — (TIF) [file pone.0139796.s001.tif]
